# Steam, and Its Effects on Rubber and Celluloid When Properly Applied

**Published:** 1885-05

**Authors:** F. W. Seabury


ARTICLE III.

### STEAM, AND ITS EFFECTS ON RUBBER AND CELLULOID WHEN PROPERLY APPLIED.

BY DR. F. W. SEABURY.

Feeling the general want of something more definite and satisfactory in the manner of manipulating rubber and celluloid, I have experimented, with the following results:

Rubber, exposed in a hot chamber, swells up and becomes porous; compressed in molds and subjected to heat, its density, after curing, is in proportion to the amount of mechanical pressure employed. When vulcanized in a water or steam-bath, pressure is then essential to density, or hardness. The element of toughness is secured by a mild, even temperature. There are several ways to attain this. The pot vulcanizer was the first. The rubber is placed in an air-tight vessel, immersed in water, and vulcanized from four to twelve hours. The sealed vessel may be immersed in a tray full of water and placed in a steam bath; this is a very mild heat. Chambers nearly surrounded by a water or steam-bath are in common use. These processes answer quite well; but rubber manufacturers have always been experimenting to get dry or super-heated steam, it being a good conductor of heat and a very mild fluid· With the water-bath vulcanizers now in use, it is impossible to distinguish a pure, clean rubber, from one of low-grade stock, loaded with dirt and pigments, When the several red dental rnbbers now on the market are vnlcanized in the usual way, in a Whitney or pot vulcanizer, they have to be marked before they are put in to vulcanize, in order to distinguish them when taken out. Intelligent experiments cannot be conducted with a Whitney vulcanizer, or with one constructed on the same principle.

The first thing to do, then, is to get a process of vulcanizing which will develop the color. It has been known for years that this could be accomplished by keeping the rubber absolutely dry, by sealing with tin-foil, baking in a hot-air chamber, or curing with super-heated steam. When rubber shrinks, it is either because it has not been properly seasoned, both after washing and after mixing, or it has been packed and vulcanized in a water-bath. The water will separate the rubber from the teeth every time, so that one can get the shrinkage only in a dry oven. Super-heated steam, applied instantly, is the perfect medium for conducting heat to rubber, because it gives the best color and preserves the elasticity of the rubber. Being a rarefied gas, the indicated is always the actual temperature for all parts of the oven, and rubber need never be burned or made granular or porous. That is to say, in order to vulcanize rubber properly, the steam generator must be separate from the vulcanizing chamber, so that the high-pressure steam can come into the chamber instantly, to produce pressure before the heat can be communicated to the rubber.

The term "super-heated steam" is a misnomer to me, and, I think, to others. I will try to explain my idea of it. Intensity of heat is measured by the number of heat units in a given space,—increasing the space diminishes the heat, and decreasing the space increases the temperature, in uniform ratio. When fire is applied to steam, the vapor atoms are expanded, and, of course, occupy a larger space, and if they are not confined their temperature will decrease. A friend of mine, Hon. Ellery Wilson, president of the Rumford Chemical Works, extended a steam-pipe through a large fire-pot, heated white hot, in order to get super-heated steam. He was surprised to feel a draft of cool air coming out of the pipe. After super-heating steam in my vulcanizer, if the test-cock is opened, the steam will feel cool. So, to my mind, "super dry steam" would more nearly express the product. George W. Richardson, the inventor of the pop safety-valve, in order to save the waste from radiation and increase the power of the steam by super-heating, jacketed a locomotive all over, including the cylinder heads, and attached a blower to the flue. The products of combustion in the flue registered 800° F.; the temperature of the steam was 400°. The hot air lost 300° in passing through the jacket, without increasing the temperature of the steam. The locomotive was run one week, using the blower, and the next week without it; coal and water were measured both weeks, and there were no difference. So, to increase the temperature one must in crease the pressure, the heat of steam being dependent on pressure. This is in accordance with the law of mutual repulsion of gases. Vapor lacks capacity for heat. A drop of water will contain 830 times more heat than a correspon-ding drop of vapor. The steam being dry, the color of the rubber is improved, and is of course uninjured by water or moisture.

To harden dental rubber appears a simple matter enough; but to do it so that it may maintain its best qualities is more difficult than most persons suppose, and a large proportion of the rubber dentures manufactured are either over-steamed or insufficiently hardened. For this there are several reasons: First, it is found that as the pressure of steam in water increases the conducting power decreases, and the heat tends to accumulate near the point of application. Second, the air in the top of the vulcanizing chamber protects the thermometer from the steam, as air, when still, is a non-conductor. Wildman, by opening a safety valve in the top of the Whitney vulcanizer, thus causing a circulation, increased the indicated pressure thirty-five pounds to the square inch. Rubber manufacturers, when vulcanizing, keep blowing off steam, thereby causing a circulation and equality of heat in all parts of their long ovens. Third, when water comes in contact with rubber, it destroys the color, makes it soft and porous, and also prevents it from adhering to the teeth.

All authorities agree that to get the best results when vulcanizing rubber the temperature of the vulcanizing chamber should be gradually and slowly raised. My process is just the opposite. I commence with dry steam, at high pressure and high temperature, and I claim that rubber cured in this way is tougher, takes a higher polish, and makes a closer union with the teeth; produces a lighter and brighter color, and requires less time to vulcanize and finish.

*How Celluloid is made.*—A roll of paper is slowly unwound, and at the same time saturated with a mixture of five parts of sulphuric acid and two of nitric, which falls on the paper in a spray. This changes the cellulose of the paper into a fine pyroxyline (gun cotton). The excess of acid having been expelled by pressure, the paper is washed with plenty of water until all traces of acid have been removed; it is then reduced to pulp, and passed on to the bleaching trough. Most of the water having been got rid of by means of a strainer, the pulp is mixed with from twenty to forty per cent. of its weight of camphor, and the mixture thoroughly triturated under millstones. The necessary coloring-matter having been added in the form of powder, a second mixture and grinding follows. The finely divided pulp is then spread out in thin layers on slabs, separated from one another by sheets of blotting paper, and from twenty to twenty-five of these layers are placed in a hydraulic press, and are subjected to a pressure of 140 atmospheres until all traces of moisture have been got rid of. The plates thus obtained are broken up and soaked for twenty-four hours in alcohol. The matter is then passed between rollers, heated to from 140° to 150° Fahr., whence it issues in the form of elastic sheets. These sheets are then molded into dental blanks, at 260°, and seasoned. Celluloid is in general use, and, as manipulated by manufacturers, the colors stand; it does not warp or crack, and there is no shrinkage. The reason of its failure for dentures is due entirely to the improper method of molding it. Celluloid will adhere to porcelain with sufficient tenacity to pull the enamel off a tooth. Molden on metal dies, and covered with tinfoil, it receives a vitreous surface, which is a non-irritant, and also protects it from the fluids of the mouth. In using single teeth on metal plate, it is necessary to have some substitute for the gum. Celluioid, when properly made, answers the purpose well. Molded in a dry chamber, at 300° Fahr., it will adhere to the gold plate and teeth, making a perfect union, and thus preventing the absorption and retention of the fluids of the mouth and consequent offensive odor. Used as a veneer on gold plate, it is easily repaired.—*New York Odontological Society Proceedings.*

